# The Improved Antineoplastic Activity of Thermophilic L-Asparaginase Tli10209 via Site-Directed Mutagenesis

**DOI:** 10.3390/biom14060686

**Published:** 2024-06-12

**Authors:** Lijuan Zhang, Simeng Ding, Xiuhui Tang, Renjun Gao, Rui Huo, Guiqiu Xie

**Affiliations:** 1 School of Pharmaceutical Sciences, Jilin University, Changchun 130021, China; zlj20@mails.jlu.edu.cn; 2 Key Laboratory for Molecular Enzymology and Engineering of Ministry of Education, School of Life Science, Jilin University, Changchun 130021, China; dingsm23@mails.jlu.edu.cn (S.D.); tangxh19@mails.jlu.edu.cn (X.T.); gaorj@jlu.edu.cn (R.G.)

**Keywords:** L-asparaginase, targeted mutagenesis, antitumor, immobilization, gold nanorods

## Abstract

Amino acid deprivation therapy (AADT) is a novel anticancer therapy, considered nontoxic and selective. Thermophilic L-asparaginase enzymes display high stability and activity at elevated temperatures. However, they are of limited use in clinical applications because of their low substrate affinity and reduced activity under physiological conditions, which may necessitate an improved dosage, leading to side effects and greater costs. Thus, in an attempt to improve the activity of L-Asn at 37 °C, with the use of a semi-rational design, eight active-site mutants of *Thermococcus litoralis* DSM 5473 L-asparaginase Tli10209 were developed. T70A exhibited a 5.11-fold increase compared with the wild enzyme in physiological conditions. Double-mutant enzymes were created by combining mutants with higher hydrolysis activity. T70A/F36Y, T70A/K48L, and T70A/D50G were enhanced by 5.59-, 6.38-, and 5.58-fold. The immobilized enzyme applied in MCF-7 breast cancer cells only required one-seventh of the dose of the free enzyme to achieve the same inhibition rate under near-infrared irradiation. This provides a proof of concept that it is possible to reduce the consumption of L-Asn by improving its activity, thus providing a method to manage side effects.

## 1. Introduction

Amino acids perform a critical role in metabolism as they are used for the synthesis of proteins, low-molecular-weight compounds, and intermediate metabolites. To adapt to their increased demands for energy, reducing substances, and cellular biosynthesis, tumor cells change their metabolic pathways, one of which manifests as an auxotrophy to specific amino acids, resulting in their sole dependence on englobing exogenous amino acids to sustain normal metabolism [1,2]. This phenomenon has made amino acid deprivation therapy (AADT) a promising method in cancer therapeutics. With the aim of limiting the supply of specific amino acids to cancer cells and thus inhibiting their growth and proliferation, AADT uses enzymatic hydrolysis to consume them [3,4]. This therapy has minimal harmful effects on normal cells due to their lower demand for amino acids when compared with cancer cells and their ability to synthesize specific amino acids under auxotrophic conditions. Asparagine, arginine, glutamine, and methionine all have application potential. To date, asparagine is the most successful application target in amino acid deprivation therapy, with an obvious effect on acute lymphoblastic leukemia (ALL) [5]. In some solid tumors, such as breast cancer, lung cancer, and ovarian cancer, asparagine mediates ALL and therefore also has therapeutic potential [6,7,8].

L-asparaginase (EC3.5.1.1) is a kind of hydrolytic enzyme that can hydrolyze L-asparagine into L-aspartate and ammonia. Adding L-asparaginase exogenously to the environment of tumor cell growth can cause cancer cell death and inhibit proliferation due to the inability to obtain essential amino acids for growth [9]. However, some side effects limit its clinical application. L-asparaginase derived from microorganisms has the advantages of economy and simplicity [10], and yet its recovery from microorganisms can be challenging and there may be immunological side effects during treatment. As L-asparaginase can accept both L-glutamine and L-asparagine as substrates, the decrease in glutamine content will initiate diseases such as pancreatitis, cerebral hemorrhage, and neurological disorders [11,12]. In addition, due to its drawbacks of a short half-life and low stability, repeated administration is required during clinical therapy, placing a burden on patients. Therefore, developing an L-asparaginase with equivalent L-asparaginase activity but with low-to-no L-glutaminase activity is a new strategy to prevent its side effects.

With resistance ability under a variety of harsh physicochemical conditions, thermophilic proteins display exceptional stability and activity at high temperatures, making them useful industrial enzymes. However, in clinical application, their benefit remains far from reach, as enzymes for therapeutic purposes should produce optimum activity under physiological conditions, but thermophilic proteins work best at high temperatures. In this context, protein engineering approaches may be used to improve thermophilic enzymes’ activity combined with their advantage of favorable stability [11]. A semi-rational design is a method for the selection of some amino acid residues for site-specific mutagenesis based on a certain understanding of the physicochemical properties, tertiary structures, and catalytic sites of the protein. For some enzymes with low structural homology to the resolved structures in the database, there is an effective and reasonable method for selecting mutation sites through multiple sequence alignment of the family, which may be used to search for beneficial mutation sites corresponding to the target protein. With the development of algorithms, computer auxiliary protein design has gradually been applied to protein modification. For enzymes whose structures have been solved, the method of molecular docking can be used to dock the tertiary structure of proteins with their ligand molecules to analyze the interaction between ligand and bound amino acids. According to the purpose of mutation, a specific amino acid is mutated to change the strength of the interaction force and thereby change the affinity between the protein and the ligand; then, positive mutants are obtained through the experiment.

In the present study, *Thermococcus litoralis* DSM 5473 L-asparaginase Tli10209, an enzyme previously cloned [13], expressed, and characterized with an optimal temperature of 90 °C, was chosen. Because of its optimal activity at high temperatures, Tli10209 has the advantages of exceptional stability, a long half-life, and low glutaminase activity at body temperature (37 °C), which reduce its impact on the body. However, the hydrolysis ability of L-asparagine also decreases under physiological conditions, and its activity decreases compared to generics, causing an increase in usage [14]. Therefore, a rational approach was adopted, and selective mutations were made to improve the enzymatic properties. Through semi-rational design, we used calculation design software BIOVIA Discovery studio 2021 for molecular docking and the determination of suitable mutated sites combined with a comparison of sequences, aiming to select improved active mutants. Afterward, in an effort to give full play to the advantages of thermophilic proteins, the method of near-infrared improved active mutants was used on gold nanoparticles, and increased antitumor activity and significantly reduced usage of enzymes of MCF-7 breast cancer cells were observed, thus highlighting the clinical application prospects of Tli10209.

## 2. Materials and Methods

### 2.1. Chemicals and Reagents

Fast Pfu DNA polymerase, Quick Cut Dpn I, and T4 DNA ligase were purchased from TransGen Biotech (Beijing, China). A Tianprep Mini Plasmid Kit and Universal DNA Purification Kit were purchased from Tiangen Biotech (Beijing, China). MCF-7 breast cancer cells were purchased from the Chinese Academy of Sciences (Shanghai, China). DMEM cell culture medium was purchased from Gibco (New York, NY, USA). FBS fetal calf serum was purchased from Maikangyuan Biotechnology (Beijing, China).

### 2.2. Strains and Vectors

*E. coli* BL21 (DE3) was employed as the host strain for protein expression. Plasmid pET-28a (+) was employed as the expression vector. Both were kept in our laboratory.

### 2.3. Experimental Methods

#### 2.3.1. Simulation and Analysis of the Tertiary Structure of L-Asparaginase

The structures of L-asparagine and L-glutamine were downloaded from the National Center for Biotechnology Information (http://www.ncbi.nlm.nih/, accessed 6 April 2022); Protein BLAST (http://blast.ncbi.nlm.nih.gov/, accessed 21 March 2022) was used to retrieve the protein sequence of *Thermococcus litoralis* DSM 5473 and predict the protein structure with the use of Alphafold (AF-H3ZJL8-F1) [15]. For analyzing conservative residues of Tli10209, a multiple-sequence alignment of L-asparaginase from different sources was conducted with Jalview 2.11.3.3. BIOVIA Discovery studio 2021 was applied to consider potential binding patterns between Asn and L-asparaginase with the method of molecule docking. Using CDOCKER, the ligand-binding cavity was defined based on the active site, setting the Top Hits and Pose Cluster Radius to 10 and 0.5. The RMSD threshold was set to 0.5 Å to ensure that the docking conformations were as diverse as possible, and the rest of the parameters were set to the default. The best docking model was judged using the scoring function CDOCKER ENERGY [16]. The highest value of CDOCKER ENERGY proved to be the best docking result for the active site in order to choose amino acids in the range of 5 Å. Subsequently, PyMoL 2.4.0 was operated to visualize the best docking model determined by BIOVIA Discovery studio 2021 [17].

#### 2.3.2. Construction of a Recombinant Plasmid of Site-Mutated L-Asparaginase

Recombinant plasmid pET 28a-Tli10209 was used as a template. Mutation primers for each mutant were designed (Table S1) and used to complete mutation through the method of whole-plasmid PCR mutagenesis during overlap extension. After confirming that the PCR was performed correctly and resorting to agarose gel electrophoresis, DpnI-treated PCR products were recovered with a Universal DNA Purification Kit and then fixed with T4 DNA ligase, and circular plasmids were transformed into competent *E. coli* BL21 (DE3) cells for expression [13]. Mutant plasmids strains were grown on LB medium plates and selected with 50 μg/mL kanamycin. Positive clones were picked out and further verified using DNA sequencing.

#### 2.3.3. Expression and Purification of Proteins

Mutant strains with correct sequences were revitalized in vitro and with an enlarged culture. When OD_600_ of the culture reached 0.6–0.8, isopropyl-β-D-thiogalactopyranoside (IPTG) at a final concentration of 0.5 mM was added to induce protein expression. The precipitant was harvested, and 1 g was resuspended in 50 mM phosphate buffer (pH 7.5) and lysed by sonication. The cellular debris was removed via centrifugation. Since the recombinant plasmid contained a His tag, the supernatant was used for protein purification with Ni^2+^-NTA. Target proteins were eluted in different concentrations of imidazole and collected for the SDS-PAGE analysis (containing 12% separate gum and 5% concentrated gum). The solution after dialysis was concentrated with the usage of polyethylene glycol (PEG). The protein concentration was determined by the Bradford method using bovine serum albumin as a standard [18]. For convenient comparisons, we used 0.1 mg/mL concentrations of the wild-type enzyme and mutants.

#### 2.3.4. Determination of L-Asparaginase Enzyme Activity

Asparaginase activity was measured by measuring the amount of NH_3_ produced, as determined with a Nessler reagent assay [19]. The reaction was carried out as follows: 200 µL of PBS (50 mM, pH 7.5) and 10 µL of pure enzyme were added, followed by 100 µL of L-Asn (80 mM), and the reaction was carried out in a water bath for 5 min. We added 100 µL of 1.5 M trichloroacetic acid (TCA) added at the end of the reaction to terminate it. Next, we added 200 µL of Nessler’s reagent, mixed well, and let it stand for 10 min at room temperature to develop color, and then we measured the absorbance at 450 nm by using a UV spectrophotometer after the color development. (The operation of the zeroing group was the same as above, except that no enzyme was added).

#### 2.3.5. Characterization of L-Asparaginase Tli10209 and Mutants

Optimum temperature determination: The reaction temperatures were set to 40 °C, 50 °C, 60 °C, 70 °C, 80 °C, 90 °C, and 95 °C, and the buffer solutions were all PBS with pH 7.5. The optimum temperature was determined according to the enzyme activity levels at different temperatures.

Optimal pH determination: Since the aim of this study was to screen for mutants with increased enzyme activity, only the mutants with increased enzyme activity in the above optimal temperature determination were subjected to optimal pH determination. The buffer systems used were as follows: sodium acetate–acetic acid buffer solution (50 mM, pH 5.0–8.0), and sodium bicarbonate buffer solution (50 mM, pH 8.0–12.0). The assay temperature was 60 °C.

Kinetic parameter determination: The kinetic parameters of L-asparaginase Tli10209 and the dominant mutants were determined using L-asparagine as the substrate, with the final concentration of the substrate ranging from 0.5 to 18.0 mM. The enzyme activity (U/mg) at different substrate concentrations was obtained at 80 °C and pH 10, after which the *K_m_* (mM) and *V_max_* (μmol·min^−1^·mL^−1^) for each mutant were calculated using GraphPad Prism 7.0. Then, *k_cat_* (s^−1^) and *k_cat_/K_m_* (mM^−1^·s^−1^) were calculated according to the formula *k_cat_* = *V_max_*/[E].

Thermal stability determination: PBS buffer solution as the comparison was placed in a differential scanning calorimeter (DSC) with Tli10209 and dominant mutants, whose concentrations were all 0.1 mg/mL. After heating up at the set rate, the DSC thermogram was obtained, with the temperature as the abscissa and the amount of heat required to be supplied when the temperature difference between the sample and the reference was zero as the ordinate. According to the curve, Tm (melting temperature) was analyzed.

Temperature stability determination: The temperature stability of L-asparaginase Tli10209 and the dominant mutant were determined using L-Asn as the substrate, and the method of determining the L-asparaginase activity was the same as in Section 2.3.4. The reaction temperatures were set to 60 °C, 70 °C and 80 °C, and 10 μL of enzyme was collected every 12 h for measurement. A value of 100% was assumed for 0 h, and relative activity–time curves were plotted according to the experimental results.

#### 2.3.6. MD Simulation

To analyze the contribution of the mutation to improving the activity of L-asparaginase, MD simulation of Tli10209 wild-type and dominant mutants K48L, T70A, D50G, and F36Y was performed. Molecular dynamics simulations were performed under the Amber ff14SB force field [20]. The proteins were initially neutralized by adding one sodium atom and then placed in a water box (8 Å distance between any protein atom and the edge of the box) [21]. Energy minimization of the protein structures was performed using the steepest descent integrator (500 steps) and a conjugate gradient algorithm (500 steps). Furthermore, we performed slow heating from 0 to 300 K of each system, and those were equilibrated under a constant volume (NVT) ensemble and a constant pressure (NPT) ensemble at 300 K for 50 ps [22], followed by MD simulation for 100 ns at 300 K. Trajectory analyses included root mean square deviation (RMSD) and root mean square fluctuation (RMSF).

#### 2.3.7. Synthesis of Gold Nanorods and Immobilization of Enzymes

Due to the unique surface plasmon resonance (SPR) properties of gold nanorods, there are two SPR peaks in the transverse and longitudinal directions. The longitudinal SPR peak depends on the gold nanorod length to short axis ratio and is continuously adjustable from visible (550 nm) to near infrared (1550 nm) [23]. This implies the ability to use the thermal energy converted by gold nanorods to take advantage of thermophilic enzymes for photothermal therapy. The steps for synthesizing gold nanorods in this paper were based on the work of Nikoobakht [24]. The crystalline seed growth method was used, where the seed solution is first synthesized and then added to the reduced growth solution at a suitable temperature to grow them overnight. Gold nanorods can exhibit unique extinction properties in the UV-visible spectrum, generally showing two peaks, near 520 nm and between 700 and 800 nm, so the prepared gold nanorods were diluted and then characterized by a full-spectral scan using the UV spectrophotometer.

After the preparation of gold nanorods, the enzyme was immobilized with the gold nanorods by selecting the mutant with higher viability. The enzyme was immobilized on the surface of the gold nanorods by using the strong interaction between the sulfur atoms in the sulfhydryl group (-SH) contained in the enzyme and the gold surface to form the enzyme–gold nanorod complex [25]. The steps were as follows: Firstly, 1 mL of the gold nanorod solution prepared above was added to the reaction flask, and 1 mg of the enzyme to be immobilized; then, the pH of the system was adjusted to 9 with 0.2 M potassium carbonate, and, finally, the system was made up to 4 mL with ultrapure water, and the enzyme was brought into full contact with the gold nanorods and immobilized in the chromatography chamber at 4 °C for 4 h. After immobilization, the precipitate was collected via centrifugation (12,000 rpm, 4 °C, 5 min) and resuspended with 1 mL of ultrapure water to obtain the immobilized enzyme. The full spectrum of the immobilized enzyme was also scanned using UV-visible spectroscopy. Theoretically, there should be a peak indicating a protein near 280 nm in addition to the two peaks of the gold nanorods, so the simultaneous presence of three peaks at 280 nm, 520 nm, and 700–800 nm indicated that the enzyme was successfully immobilized on the gold nanorods.

#### 2.3.8. Cell Proliferation Inhibition Assay

The effect of L-asparaginase Tli10209 and its mutant free enzyme and immobilized enzyme on the proliferation ability of MCF-7 breast cancer cells was evaluated using the MTT method [26]. MCF-7 cells were inoculated into 96-well plates at 4000 cells/well, and after the cells were plastered, the original medium was replaced with media containing different concentrations of free enzyme or immobilized enzyme (three replicate wells were set up for each group), and after 6 h, the immobilized enzyme medium was discarded and the cells were washed with PBS to prevent the color of the immobilized enzyme from affecting the absorbance measurement. We added 100 μL medium (containing 10% FBS) in each well and then irradiated the positive control group with an NIR emitter (2 W for 5 min per well), while the free enzyme group was left without this treatment. The cell viability was calculated according to the following formula:

Cell viability (%) = (sample OD − blank OD)/(control OD − blank OD) × 100%(1)

**Theorem** **1.** *Theorem of cell viability. The OD of the sample group is the OD of the enzyme-containing group; the OD of the control group is the OD of the medium serum-only group; the OD of the blank is the OD of the DMSO-only group.*

## 3. Results

### 3.1. The Selection of an Improved Active Site-Mutation of L-Asparaginase

**Figure 1:**
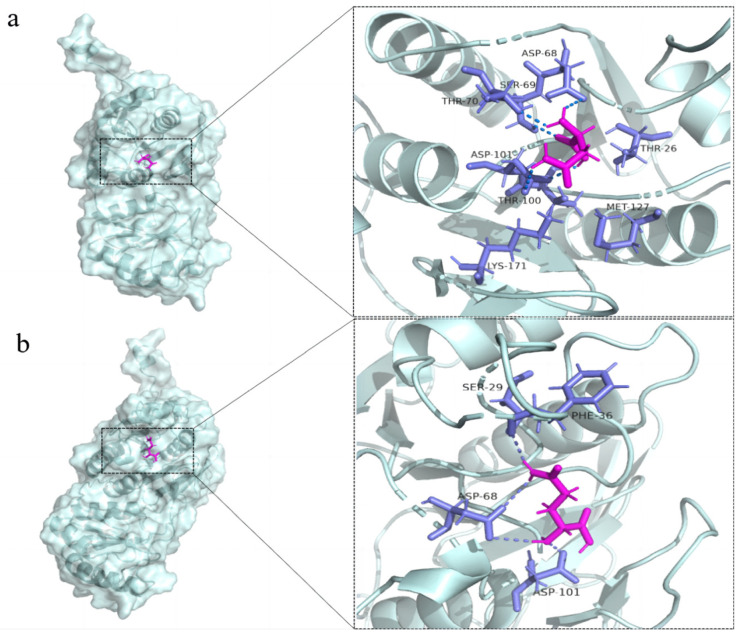
Results of molecular docking of Tli10209 (gray) with L-asparagine (**a**) and L-glutamine (**b**). Substrates L-asparagine and L-glutamine are indicated in magenta. Amino acids in the 5 Å range of L-asparagine and L-glutamine are indicated in purple.

L-asparaginase Tli10209 originating from *Thermococcus litoralis* DSM 5473 is an enzyme with 345 amino acids encoded by 1038 basic groups (AF-H3ZJL8-F1). The tertiary structure is shown in Figure 1. The results of molecular docking of Tli10209 with asparagine and glutamine display that most substrate-interactive amino acids of this enzyme are consistent with the reported catalytic sites of L-asparaginase from *E. coli*, indicating that their catalytic pockets are in accordance (Figure 1). Consequently, it seems that previously reported mutant sites of L-asparaginase from *E. coli* have reference value to modify Tli10209. Constructing Q59L, Q59D, Q59A [27], and N248S [28,29] of *E. coli* L-asparaginase significantly reduced L-glutamine activity. According to sequence alignment between Tli10209 and *E. coli* L-asparaginase, the sites are, respectively, 70 and 255 in Tli0209. Therefore, the mutant enzymes T70L, T70D, T70A and G255S were created. In *E. coli*, 25Y is the constituent amino acid in the reported catalytic triad I. At the same time, other thermophilic sources of L-asparaginase sequences with the same positional amino acid are all tyrosine. On the basis of this result, F36Y was built. In addition, to explore whether tyrosine is irreplaceable in this position, F36 was also mutated to alanine.

**Figure 2:**
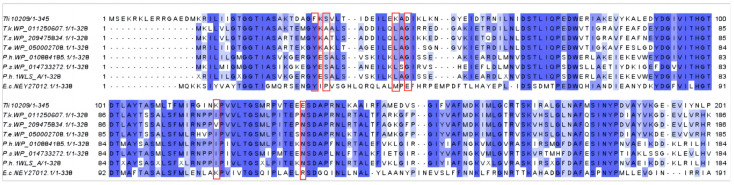
Amino acid sequence similarity of Tli10209 with other sources of L-asparaginase. Numbers on the right are the residue numbers of the last amino acid in each line. Sequences listed include L-asparaginases from *Thermococcus kodakarensis* (WP_011250607.1), *Thermococcus stetteri* (WP_209475834.1), *Thermococcus eurythermalis* (WP_050002708.1), *Pyrococcus* sp. ST04 (WP_014733272.1), *Pyrococcus horikoshii* (WP_010884185.1), *Pyrococcus horikoshii* (1WLS_A), and *Escherichia coli* (NEY27012.1). The locations of six substitutions (F36, S38, K48, D50, K119 and E134) are boxed in red.

The results of a sequence comparison of Tli10209 with other highly active thermophilic L-asparaginase, carried out in Jalview 2.11.3.3, indicated that S38, K48, D50, K119, and E134 had significant differences between these sequences (Figure 2). Due to their conservative nature, mutating these sites can maintain the integrity and function of the macromolecules. Therefore, S38A, K48L, D50G, K119I, and E134N were constructed. The HotSpot Wizard (https://loschmidt.chemi.muni.cz/hotspotwizard/, accessed on 27 March 2022) selects non-conserved amino acids located around the active site or in substrate entry/exit channels as modifiable “hotspots” by integrating structural, functional, and evolutionary information from many databases, which can be used for protein-directed design [30]. When we uploaded Tli10209 to this website, the mutation of serine at position 38 to proline was recommended based on evolutionary relationships and activity. Consequently, S38P was constructed.

### 3.2. Construction, Expression and Purification of Single Mutants of Tli10209

**Figure 3:**
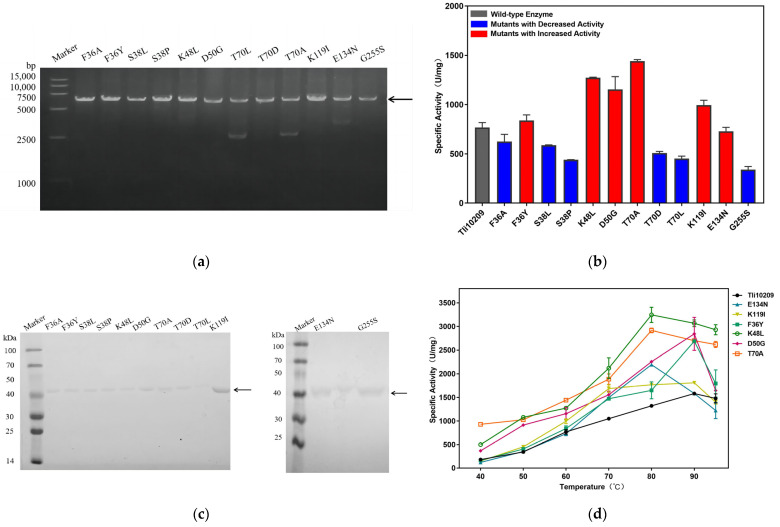
Single mutant construction, purification and characterization of Tli10209. (**a**) Agarose gel electrophoresis of the mutation PCR; (**b**) Comparison of enzyme activity between wild enzyme and single mutants at 60 °C. The wild enzyme activity was marked as gray. Mutants with higher activity than wild enzyme were marked in red. Mutants with lower activity than wild enzyme were marked as blue; (**c**) SDS-PAGE of purified mutants; (**d**) Research on optimum temperature of Tli10209 and forward mutants. (All assays were performed in triplicate, the standard deviations of the biological replicates are represented by error bars and purpose bands are indicated by arrows). Original images can be found in Figure S2.

Recombination plasmid pET 28a-Tli10209 was used as a template to propagate DNA segments based on pairs of primers (Figure 3a). The PCR product was digested and transferred into the *E. coli* BL21 (DE3) competent cells for competent heterologous expression. Mutants confirmed by DNA sequencing were purified using Ni^2+^-NTA affinity chromatography (Figure S1). A single intense band of 38.5 kDa on SDS-PAGE indicated a successfully purified protein (Figure 3c), and we found that approximately 1 mg of pure enzyme can be recovered from 1 g of precipitation.

### 3.3. Determination of Mutants’ Specific Activity

Considering that the local temperature of immobilized enzymes is approximately 60 °C after near-infrared (NIR) irradiation, the activities of the wild enzyme and mutants were measured at 60 °C (Figure 3b). When compared with the wild enzyme Tli10209, the activities of K48L, T70A, D50G, K119I, and F36Y were increased, and T70A had a 3.27-fold increase. Additionally, we analyzed the properties of these enzymes, and the optimal temperature results are shown in Figure 3d. The specific activity levels of K48L, T70A, and D50G made dramatic advances in the temperature range under consideration. Since the reactive properties of F36Y increased little at an intermediate temperature but improved obviously at 90 °C, we surmise that it has application potential.

**Figure 4:**
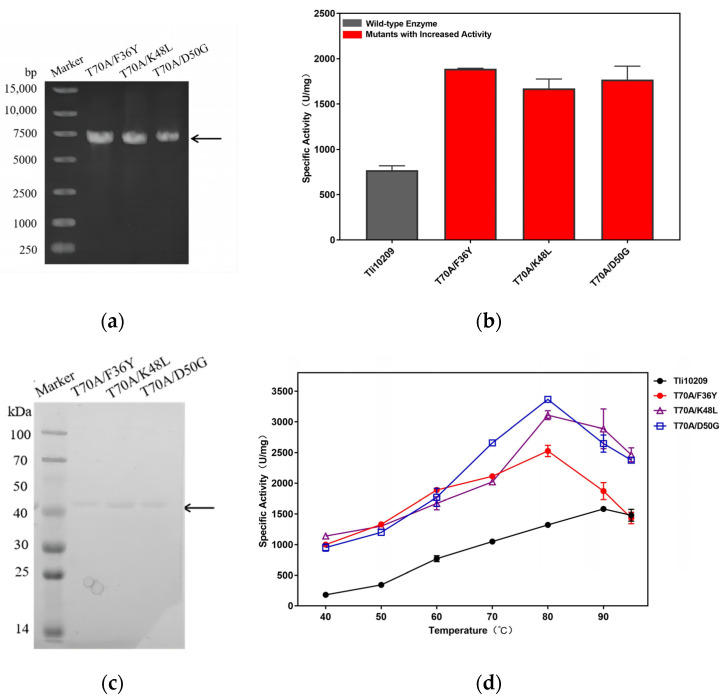
Double-mutant construction, purification and characterization of Tli10209. (**a**) Agarose gel electrophoresis of the mutation PCR; (**b**) Comparison of enzyme activity between wild enzyme and double mutants at 60 °C. The wild enzyme activity was marked as gray. Mutants with higher activity than wild enzyme were marked in red; (**c**) SDS-PAGE of purified mutants; (**d**) Research on optimum temperature of Tli10209 and forward mutants. (All assays were performed in triplicate, the standard deviations of the biological replicates are represented by error bars and purpose bands are indicated by arrows). Original images can be found in Figure S2.

To further enhance the conversion rate of mutant enzymes, double-mutant enzymes were created by combining mutants of K48L, F36Y, D50G, and T70A, which have a higher hydrolysis activity. The double-mutant enzymes T70A/F36Y, T70A/K48L, and T70A/D50G were prepared, with same methods of construction and characterization. An electropherogram of the double-mutant enzymes indicated that they were successfully constructed and purified (Figure 4a,c). These double mutants’ activity levels were all higher than those of the wild enzyme and single mutants, and for T70A/F36Y, there was a 3.57-fold increase in activity over the wild-type enzyme. The mannanase activity levels of T70A/F36Y, T70A/K48L, and T70A/D50G were enhanced by 6.64-, 6.37-, and 5.58-fold when compared with the wild-type enzyme at 40 °C (Figure 4b,d), presenting the great application potential under physiological or near-infrared conditions.

### 3.4. Kinetic Analysis of Mutant Enzymes

To explore the impact of positive mutations with enhanced specific activity on substrate recognition, the affinity of the substrate L-Asn to each site and its conversion rate were examined, along with the enzymatic efficiency. The kinetic constants for the wild-type enzyme and mutant enzymes are shown in Table 1. The value of the Michaelis–Menten constant *K_m_* quantifies the affinity of the enzyme and substrate; the larger the *K_m_*, the smaller the affinity. The *K_m_* of all single mutants was increased at different degrees, demonstrating that the majority of mutants reduced the affinity of L-Asn. The maximal velocity (*V_max_*) and catalytic constant of the mutants were higher than those of Tli10209. However, the *k_cat_* results for K48L and T70A were increased by 220% and 180%. Moreover, the catalytic efficiency (*k_cat_*/*K_m_*) of the mutants was enhanced by about 1.5-fold. The results of the present study indicated that each mutation contributed to the increased reaction rate, which made up for the negative effect of the low affinity, with the comprehensive performance of the enzyme activity enhanced.

In comparison with the wild-type enzyme, the mutant enzymes T70A/F36Y and T70A/K48L showed a 1.4-fold improvement in the catalytic efficiency (*k_cat_*/*K_m_*) for the substrate L-Asn, with a weak decrease in the substrate-binding affinity (*K_m_*) for all substrates. These results suggested that the reasons for the improved specific activity of double mutants are a reinforced affinity and an improved catalytic efficiency. The *K_m_* values of T70A/D50G reduced greatly, and the catalytic constants’ *k_cat_*/*K_m_* values increased slightly in comparison to the wild enzyme, resulting in a 2.25-fold increase and rising above all single mutants, which explained the significant improvement in the enzyme activity of T70A/D50G during the determination of the optimal temperature.

### 3.5. Characterization of Enzyme

**Figure 5:**
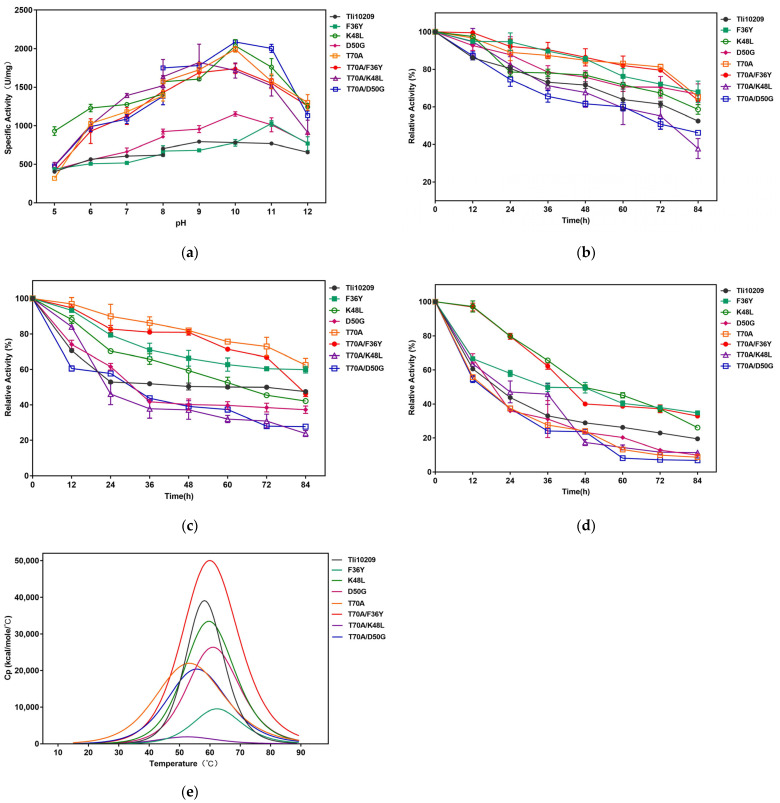
Determination of optimal pH and stability of Tli10209 and the variants derived using site-directed mutagenesis. (**a**) Research on optimum pH of Tli0209 and positive mutants; (**b**) Temperature stability of wild-type and mutants under 60 °C, (**c**) 70 °C, (**d**) 80 °C; (**e**) DSC records of heating of enzymes and dependence of heat capacity at constant pressure (Cp) on temperature for enzymes. (All assays were performed in triplicate, and the standard deviations of the biological replicates are represented by error bars).

pH is an important factor affecting enzyme activity, and suitable pH values may have a promoting effect on the enzymatic reaction. In comparison to Tli10209, the optimal pH value of all mutants was increased (Figure 5a). F36Y had the highest activity in a strong alkali condition (pH 11.0), while other mutants were the most active in weakly alkali conditions (pH 10.0). The activities of T70A and K48L were 2.57- and 2.11-fold higher than that of the wild-type enzyme at a physiological pH. The optimal pH values of Tli10209 and T70A/K48L were both 9.0, indicating that this superposition mutation had little effect on the optimum pH of the enzyme reaction. The two buffers chosen for the determination of the optimum pH were different from those used for the optimum temperature determination. Based on the results of the experiment, we found that although the mutants increased by different degrees in different buffers, the overall increasing trend was the same. Although a PBS buffer is not the optimal buffer for asparaginase, it was used in subsequent experiments because it is isotonic with human blood and is highly suitable for cell culture experiments.

As shown in Figure 5b–e, the stability levels of positive mutants with Tli10209 were compared. After normalizing the sample database by excluding referents according to concentration, records were created, and we subtracted the baseline data. The Tm values of the wild enzyme and mutants were obtained by using curve-fitting models in Origin. The residual activities of Tli10209 and the mutants were measured at 60 °C, 70 °C, and 80 °C, and their half-lives were calculated on the basis of relative activity–time curves. The results for thermal stability and temperature stability were largely consistent. The stability of most single mutants was better than that of the wild enzyme, with the exception being T70A, in accordance with the results of single mutants’ testing. The Tm and half-life of F36Y showed the highest values, and we found that overlaying T70A and F36Y could make up for the shortcoming of T70A’s low stability, which meant that the Tm of T70A/F36Y improved by 4 °C.

### 3.6. Structural Simulation and Analysis of L-Asparaginase

**Figure 6:**
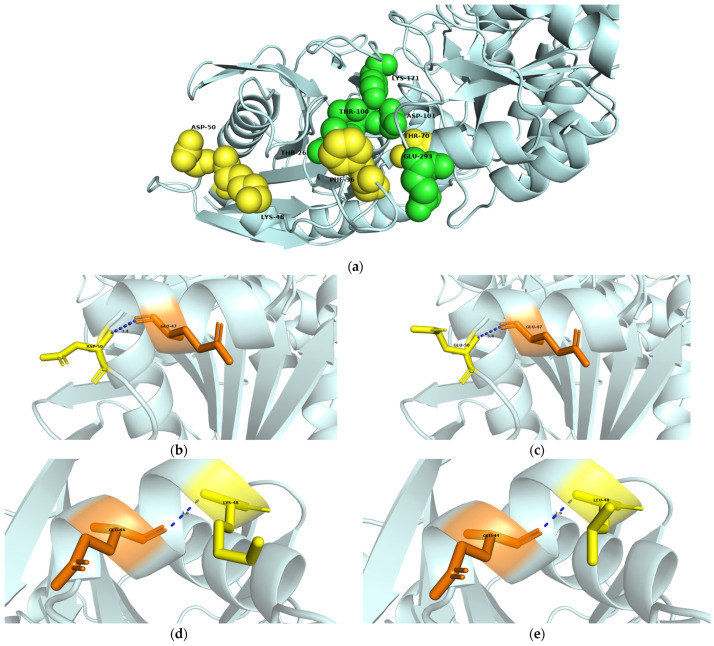

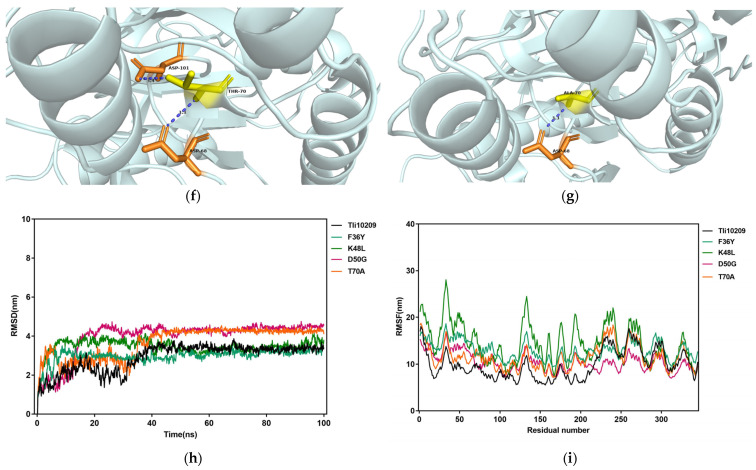
Visualization of Tli10209 three-dimensional structure, amino acid interaction forces and molecular dynamics simulation. (**a**) Three-dimensional structure of Tli10209. The spheres highlight the enzyme active sites (green) and mutation sites (yellow), as expanded in the inset; Comparison of protein structures (the short blue line represents the distance of two amino acid residues) for (**b**) Asp50, (**c**) Gly50, (**d**) Lys48, (**e**) Leu48, (**f**) Thr70, (**g**) Ala70; (**h**) root mean square deviation (RMSD) of wild-type enzyme and mutants; (**i**) root mean square fluctuation (RMSF) of wild-type enzyme and mutants.

To study the effect of the mutations on the Tli10209 structure, MD simulations were carried out on the modeled structure of Tli10209 and its mutants (Figure 6). As we found in the tertiary structure of the protein, K48 is located in the α helix, F36 and T70 are situated in the β sheet, and D50 is located in a random coil. After mutating these sites, K48L, F36Y, T70A, and D50G are still located in the α helix, β sheet, and random coil, which means the positions have no marked changes. The distance between Lys48 and Glu44 was shortened from 3.0 to 2.9 after mutation. The structural simulation results showed that there was interaction between Thr70 and both Asp68 and Asp101. The distance with Asp68 was not changed; however, the interaction with Asp101 disappeared after mutation from Thr70 to Ala70.

The dynamic behaviors of wild-type and mutant systems were analyzed through 100 ns of MD simulations, where RMSD characterized the stability of the enzyme. The results showed that all the structures reached an equilibrium after 100 ns of simulations, which indicated that the simulated trajectories of 50 ns to 100 ns were stable and representative. The RMSF values of F36Y exhibited smaller fluctuations, and the decreased RMSF values explained why the mutants are more thermostable. The RMSF values of K48L and D50G were similar to those of the wild-type enzyme. The mutant T70A showed large fluctuations in its RMSF values, revealing that the reason for improved enzyme activity is that a more flexible whole enzyme may lead to improved catalytic efficiency.

### 3.7. Synthesis and Enzymatic Immobilization of Gold Nanorods

**Figure 7:**
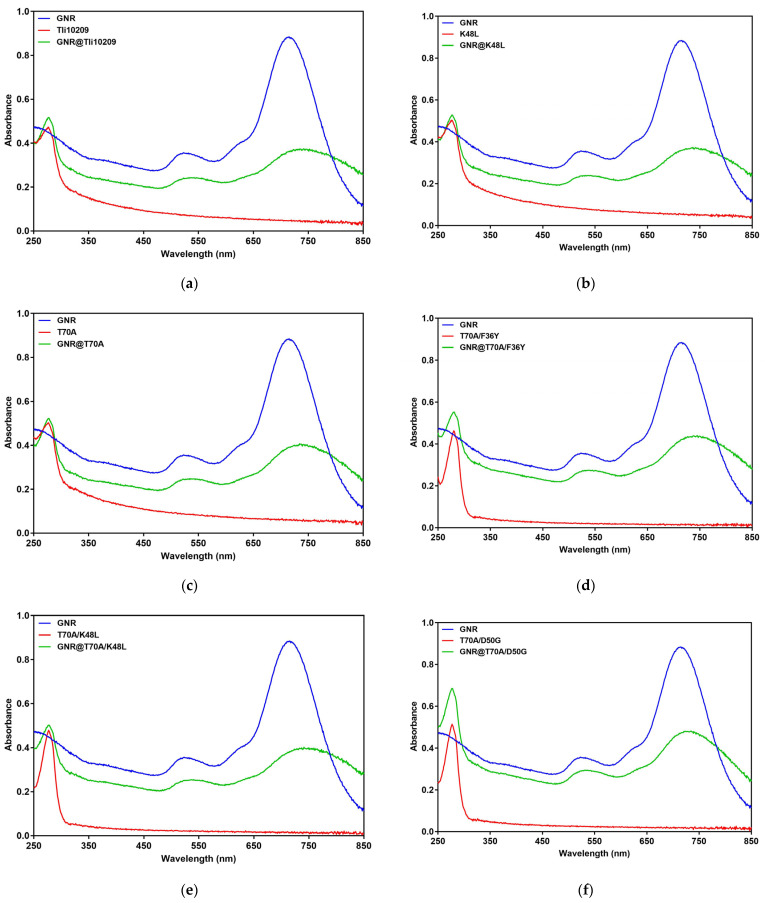
UV-Vis of free and immobilized enzymes (**a**) Tli10209, (**b**) K48L, (**c**) T70A, (**d**) T70A/F36Y, (**e**) T70A/K48L, and (**f**) T70A/D50G. (The blue line indicates gold nanorods, the red line indicates the enzyme, and the green line indicates the immobilized enzyme).

Based on these experimental results, the mutants K48L, T70A, T70A/D50G, T70A/K48L, and T70A/F36Y, which improved the activity significantly, were chosen to be immobilized on the gold nanorods, with the aim of leveraging the advantages of thermophilic enzymes and achieving better results by increasing the local temperature in near-infrared irradiation. The full spectrum of the newly synthesized gold nanorods was scanned using a UV spectrophotometer at 250–850 nm, and the results are shown in Figure 7. The synthesized gold nanorods have absorption peaks at 523 nm and 715 nm, which is consistent with the expected results. The wild enzyme and the mutants were immobilized on the gold nanorods, and the full spectrum of the immobilized enzymes (green line) was scanned and compared with the spectral results of the free enzyme and the gold nanorods. The spectral results of the immobilized enzyme showed that both absorption peaks were fused, indicating that the enzyme was successfully immobilized with the gold nanorods. Under near-infrared irradiation, the activity of immobilized enzymes was higher than that of wild-type enzymes; that of T70A/F36Y was three-fold higher than that of Tli10209 at 37 °C (Figure S3).

### 3.8. Cell Proliferation Inhibition Assay

**Figure 8:**
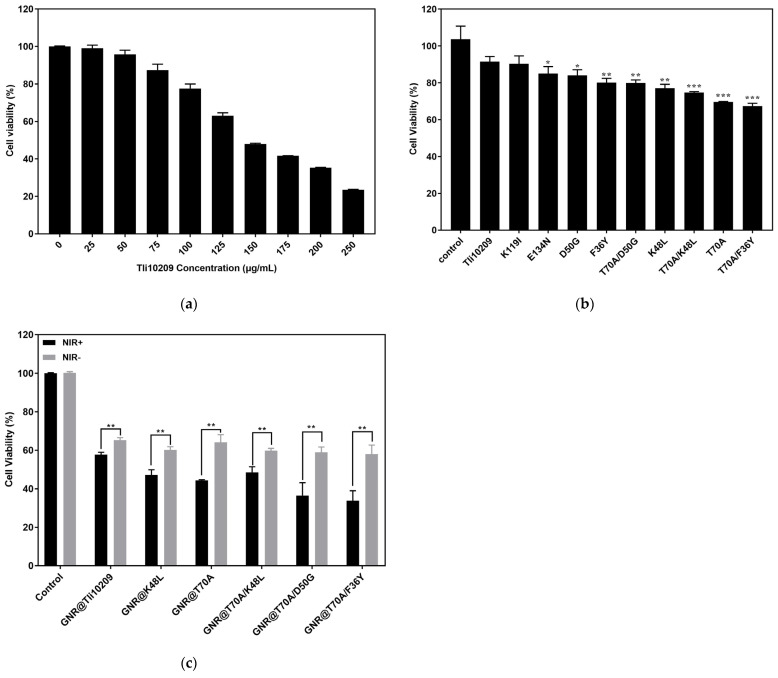
Validation of proliferation inhibition in wild-type and mutant cells. (**a**) Proliferation inhibition of breast cancer cells MCF-7 by different concentrations of Tli10209; (**b**) Proliferation inhibition of breast cancer cells MCF-7 by different free mutants; (**c**) Proliferation inhibition of breast cancer cells MCF-7 by immobilized mutants before and after NIR activation. (Statistical significance of differences between groups by one-way ANOVA using GraphPad Prism 7.0 (* *p* < 0.05; ** *p* < 0.01, *** *p* < 0.001.)

The results for the proliferation inhibition of MCF-7 breast cancer cells by different concentrations of free wild-type enzymes are shown in Figure 8a. The inhibition of cell proliferation by Tli10209 showed an obvious concentration dependence, where the anti-tumor effect was largely consistent with activity. The strongest inhibition of cell proliferation by T70A/F36Y, with a cell survival rate of 66.4% (compared to a survival rate for the wild type of 91.5%), was reduced by 25.1%, which means that the amount of enzyme required to achieve the same treatment effect as wild-type T70A/F36Y would be greatly reduced.

Compared with the free enzyme at 125 μg/mL, the concentration of 25 μg/mL of immobilized enzyme could achieve the same inhibition [13]. Immobilized enzymes of T70A/D50G, T70A/K48L, and T70A/F36Y had a more significant inhibitory effect on cell proliferation after NIR activation. The cell survival rate after NIR activation was only 35% when compared with the control group (Figure 8c), though achieving a better effect than that after free enzyme immobilization and activation at 75 μg/mL, where the usage decreased by one eighth. Other mutant immobilized enzymes also showed higher cell proliferation inhibition than the wild type, indicating that immobilized enzymes show superior effects to those of free enzymes in tumor treatment due to the dual effect of photothermal and amino acid deprivation therapy.

## 4. Discussion

The usage of L-asparaginase in adult cancer populations has been hampered because of its toxicity profile, including immunological side effects and non-immune related toxicities. After being injected into bodies as a therapeutic protein, normal cells and leukemia lymphoblasts can be cleaved by lysosomal proteases (cathepsin B, CTAB) and asparagine endopeptidase (AEP) to enhance antigen processing and promote immune reaction. Therefore, arresting proteases degrading L-ASNase is one of the main tactics to reduce immunological side effects. Maggi M mutated the specific amino acid residues cleaved by AEP and obtained mutant N24S, showing preserved activities, improved thermal and proteolytic resistance, and long-term cytotoxicity in vitro [31]. Furthermore, an enzyme coupled with polyethylene glycol can also reduce immunological side effects, and in this regard, commercial PEG-L-ASNase has already been proposed. Non-immune-related toxicities are lower in frequency but may develop a higher severity, caused by the residual glutaminase co-activity of L-ASNases, which hydrolyze Gln to Glu and ammonia, representing an important factor contributing to liver dysfunction and neurotoxicity [11,12]. As such, it is of great significance to modify L-ASNases to be more preferable for asparagine.

In this study, the appropriate mutation sites were selected through a semi-rational design, combined with molecular docking and sequence alignment. The dominant mutants with improved activity—T70A, K48L, D50G, F36Y, K119I, and E134N—were screened and characterized. The specific activity of T70A, K48L, D50G, and F36Y made significant advances in the temperature and pH range under consideration. The kinetic parameters showed that their improved activities were caused by the increasing rate of substrate conversion and the enzyme catalytic rate. The length of the hydrogen bond between Lys48 and Glu44 was longer, and the small side chain of leucine reduced the space limit of substrate entry and exit, while easing the substrate entry and exit. Alongside the increased flexibility of RMSF displayed, these factors may also allow Thr34 to rotate to an active conformation with less steric hindrance, which improves enzyme activity. According to the tertiary structure and MD simulations (Figure 6), the mutation of the 36th amino acid site from phenylalanine to tyrosine allowed the benzene ring to have an additional hydroxyl group, making it easier to accept protons during the nucleophilic attack on the enzymatic reaction, which improves the enzymatic reaction rate. Similar to K48L, Gly50 was hydrophobic, and since both sites are on the surface of the protein, the enhanced hydrophobicity made the structure more reasonable and stable. Since T70 mutated to alanine, the interaction with D101 disappeared and no hydrogen bond was formed, making it easier to accept hydrogen ions, which promoted the reaction to continue, since D101 was a catalytic active site.

Then, the dominant mutants for superposition mutation, T70A/D50G, T70A/K48L, and T70A/F36Y, were constructed and characterized. At a medium temperature, the activity of the double mutants increased more than that of the single mutation, with the enzyme catalytic rate increased according to kinetic parameters. In order to verify whether the anti-tumor activity of mutants with increased enzyme activity was as expected, breast MCF-7 cancer cells were used for verification. The results showed that the dominant mutants, except for K119I and E134N, had greatly improved proliferation inhibition; T70A/F36Y and T70A had the best effects, followed by K48L, F36Y, and D50G, consistent with our expectations.

In addition to a semi-rational research design, real-time activation including near-infrared (NIR), alternating magnetic field (AMF), microwave, and ultrasound irradiation can also remotely and spatiotemporally improve enzyme activity and maximize the enzymes’ biological function. Accordingly, this is applied in various fields, such as cancer therapy, the food industry, and environmental engineering. The universal NIR-activated strategy involves the combination of enzymes and plasmonic nanoparticles using immobilization methods such as cross-linking, physical adsorption, and encapsulation [32]. Under NIR, the LSPRs of plasmonic nanoparticles can transform optical energy into thermal energy, which leads to an elevated local temperature on the nanoparticle surface without affecting the overall temperature. Since the L-asparaginase Tli10209 originates from thermophilic bacteria, it has good stability and activity at high temperatures. Accordingly, photothermal conversion can give full play to its advantages and further enhance the enzyme activity. Therefore, the mutants K48L, T70A, T70A/D50G, T70A/K48L, and T70A/F36Y, which improved the activity significantly, were chosen for immobilization on the gold nanorods, using these experimental results. The activity of the immobilized enzymes improved evidently when compared with that of the free enzymes under near-infrared irradiation at 37 °C. The activity of T70A/F36Y was 2.91-fold higher than that of its free enzyme, while the activity levels of the other mutants were at least 2-fold higher than those of their free enzymes. The immobilized enzymes were applied in MCF-7 breast cancer cells at a concentration of one-third of the free enzymes, and their proliferation inhibition was two times that of the free enzymes.

## 5. Conclusions

In order to improve the activity of L-asparaginase Tli10209 from *Thermococcus litoralis* DSM 5473, this study selected mutation sites through sequence comparison and molecular docking. Among a total of fifteen mutants, including superimposed mutants, nine had higher enzyme activity than the wild type; the activities of T70A/D50G, T70A/F36Y, T70AK48L, T70A, and K48L were 5.58-, 5.59-, 6.38-, 5.11-, and 2.67-fold higher than those of the wild-type enzyme at a medium temperature. To play to the advantages of thermophilic enzymes, based on the immobilization models and methods available in our laboratory, mutants with greatly improved activity were immobilized with gold nanorods. The effects of free and immobilized enzymes on the inhibition of MCF-7 proliferation in breast cancer cells were measured. The cell survival rate after NIR activation was only 35% compared with the control group under the treatment with a 25 μg/mL concentration, achieving a two-fold reduction compared to that after free enzyme immobilization and activation at 75 μg/mL. Reducing the immobilized enzyme dosage lowered the cost, immunological side effects, and non-immune-related toxicities. Meanwhile, since the high-temperature pretreatment of foods such as French fries can reduce the formation of acrylamide, lowering its cancer risk, the mutants’ stability at many temperatures means they have good potential for applications in food at high temperatures. T70 and K48 mutants are significantly more vigorous than others, so a meaningful research direction will be to attempt to form these mutants with other types of amino acids, to study the mechanism of action and obtain more vigorous mutants.

## Figures and Tables

**Table 1:** The kinetic parameter of Tli0209 and its mutants.

Title 1	*K_m_* (mM)	*k_cat_* (s^−1^)	*V_max_* (μmol·min^−1^·mL^−1^)	*k_cat_*/*K_m_* (mM^−1^·s^−1^)
Tli10209	6.78	2304.55	359.3	339.90
F36Y	7.00	2502.10	390.1	357.34
K48L	9.62	5077.32	791.6	527.84
D50G	9.03	3887.53	606.1	430.70
T70A	7.90	4145.37	646.3	524.73
T70A/F36Y	6.28	2926.71	456.3	465.74
T70A/K48L	6.65	2762.51	430.7	415.29
T70A/D50G	2.68	2047.35	319.2	763.94

## Data Availability

Data are contained within the article and Supplementary Materials.

## Supplementary Materials

The following supporting information can be downloaded at: https://www.mdpi.com/article/10.3390/biom14060686/s1, Figure S1. Comparison of mutant sequencing results; Figure S2. Original images of mutant construction and purification; Figure S3. Relative activity of Tli10209 and mutants after near-infrared irradiation at 37 °C.; Table S1: Main primers used in this study.
