# Supplementary material for: The Improved Antineoplastic Activity of Thermophilic L-Asparaginase Tli10209 via Site-Directed Mutagenesis
Source: Biomolecules. 2024 Jun 12;14(6):686. doi: 10.3390/biom14060686 (PMC11202230; doi:10.3390/biom14060686)
Supplement: Supplementary file 1 [file biomolecules-14-00686-s001.zip › biomolecules-3041855-supplementary.pdf]

biomolecules

MDPI

Supplementary Materials

The Improved Antineoplastic Activity of Thermophilic L-Asparaginase Tli10209 via Site-Directed Mutagenesis

Lijuan Zhang [1,†], Simeng Ding [2,†], Xiuhui Tang [2], Renjun Gao [2], Rui Huo [1,*] and Guiqiu Xie [1,*]

[1] School of Pharmaceutical Sciences, Jilin University, Changchun 130021, China; zlj20@mails.jlu.edu.cn
[2] Key Laboratory for Molecular Enzymology and Engineering of Ministry of Education, School of Life Science, Jilin University, Changchun 130021, China; dingsm23@mails.jlu.edu.cn (S.D.); tangxh19@mails.jlu.edu.cn (X.T.); gaorj@jlu.edu.cn (R.G.)
* Correspondence: huorui@jlu.edu.cn (R.H.); jiegq@jlu.edu.cn (G.X.); Tel.: +86-13180890093 (G.X.)
† These authors contributed equally to this work.

Table S1. Main primers used in this study.

Primers	Sequences（5'-3'）
F36Y-up	GCCGGATATAAAAGCGTCCTCACAATA
F36Y-down	TTTATATCCGGCATCGGTTTTTGCG
F36A-up	GCCGGAGCAAAAAGCGTCCTCACAAT
F36A-down	TTTTTGCTCCGGCATCGGTTTTTGCG
S38L-up	TTAAACTGGTCCTCACAATAGATGAGATCC
S38L-down	TGAGGACCAGTTTAAATCCGGCAT
S38P-up	TTAAACCGGTCCTCACAATAGATGAGATCC
S38P-down	TGAGGACCGGTTTAAATCCGGCAT
K48L-up	CTTGAACTGGCTGACATAAAGCTG
K48L-down	GTCAGCCAGTTCAAGGATCTCATC
D50G-up	AAGCTGGCATAAAGCTGAAAAACGGT
D50G-down	GCTTTATGCCAGCTTTTTCAAGGATCT
T70A-up	GACAGCGCCCTTATTCAGCCCGA
T70A-down	AAGGGCGCTGTCGATATTTAAAATGTTC
T70L-up	GACAGCCTGCTTATTCAGCCCGA
T70L-down	GAATAAGCAGGCTGTCGATATTTAAAATG
T70D-up	GACAGCGATCTTATTCAGCCCGA
T70D-down	TAAGATCGCTGTCGATATTTAAAATGTTCC
K119I-up	TAACATTCCCGTGGTTTTAACTGGT
K119I-down	ACGGGAATGTTAATGCCTCTAATCA
E134N-up	TGAGGAAAATAGTGACGCCCCAAGA
E134N-down	CGTCACTTTCATTCTCAGTAACTGGGC
G255S-up	CTGGGAGCATTCCCTATAGAAAGAGAG
G255S-down	GGAATGCTCCCAGCTCCGTAACCT

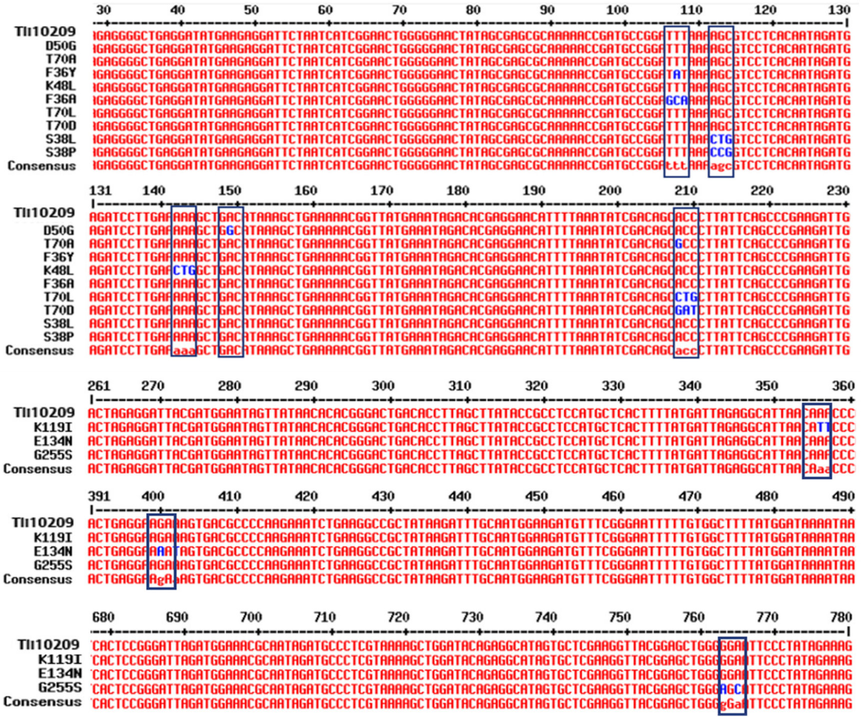

Figure S1. Comparison of mutant sequencing results. Same amino acids were marked in red. Mutant sites were marked as blue.

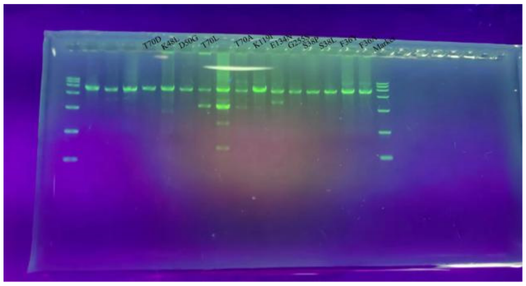

(**a**)

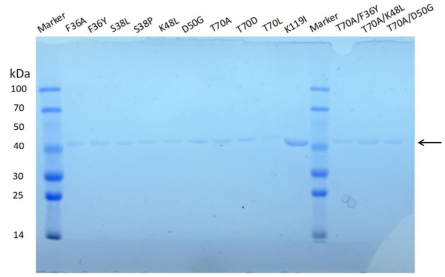

(**b**)

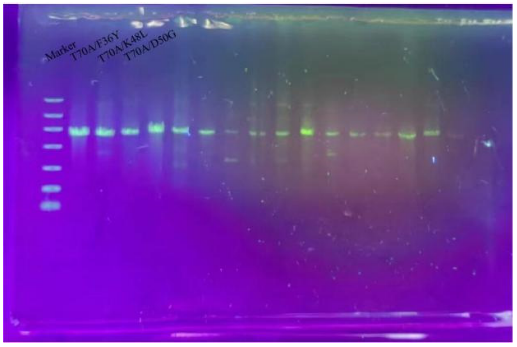

(**c**)

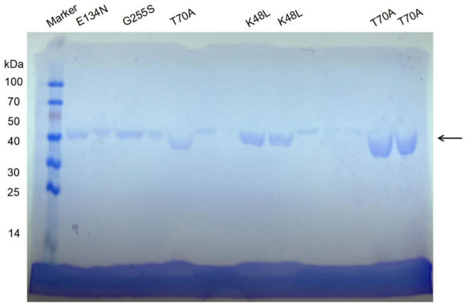

(**d**)

Figure S2. Original images of mutant construction and purification. (**a**) Agarose gel electrophoresis of the single mutation PCR; (**b**) SDS-PAGE of purified mutants; (**c**) Agarose gel electrophoresis of the double mutation PCR; (**d**) SDS-PAGE of purified single mutants. (Purpose bands are indicated by arrows).

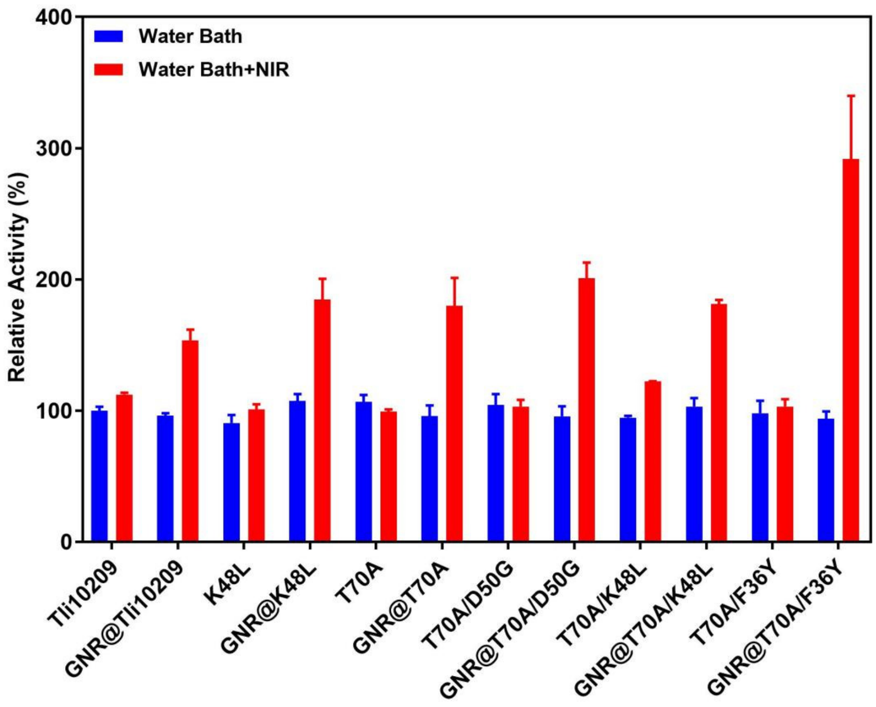

Figure S3. Relative activity of Tli10209 and mutants after near-infrared irradiation at 37℃. The specific activity of free Tli10209 was taken as 100 %. Samples without NIR were marked in blue.
